# The Brief COPE: Measurement Invariance and Psychometric Properties among Community and At-Risk Portuguese Parents

**DOI:** 10.3390/ijerph18062806

**Published:** 2021-03-10

**Authors:** Cristina Nunes, Javier Pérez-Padilla, Cátia Martins, Pedro Pechorro, Lara Ayala-Nunes, Laura I. Ferreira

**Affiliations:** 1 Psychology Research Centre (CIP) & Universidade do Algarve, Campus de Gambelas, 8005-139 Faro, Portugal; csmartins@ualg.pt; 2 Faculty of Humanities and Education Sciences, University of Jaen & Research Group (HUM604) Development of Lifestyles in the Life Cycle and Health Promotion of University of Huelva, Campus de las Lagunillas, 23071 Jaén, Spain; jppadill@ujaen.es; 3 School of Psychology, Universidade do Minho, 4710-057 Braga, Portugal; ppechorro@gmail.com; 4 Department of Psychiatry, University of Oxford, Oxford OX3 7JX, UK; lara.ayalanunes@psych.ox.ac.uk; 5 Department of Psychology and Educational Sciences, Campus de Gambelas, Universidade do Algarve, 8005-139 Faro, Portugal; liferreira@ualg.pt

**Keywords:** Brief COPE, coping, parenting, validation, psychometry, family assessment, instrumental study

## Abstract

Parenting generally brings about high internal and external demands, which can be perceived as stressful when they exceed families’ resources. When faced with such stressors, parents need to deploy several adaptive strategies to successfully overcome these challenges. One of such strategies is coping, an important cognitive and behavioural skill. In this study, we intended to examine the psychometric properties of Carver’s (1997) Brief COPE (Coping Orientation to Problems Experienced), extending its cross-cultural validity among a Portuguese sample of community and at-risk parents. The sample comprised community (*n* = 153) and at-risk (*n* = 116) parents who completed the brief COPE, the Family Adaptability and Cohesion Scales and the Parenting Stress Index—Short Form. Confirmatory factor analysis, internal consistency, cross sample invariance, convergent and discriminant validity were analysed. Data from the confirmatory factor analysis revealed that the 14-factor model obtained the best fit. The results provided evidence that the Brief COPE is a psychometrically sound instrument that shows measurement invariance across samples and good reliability. Our findings demonstrated that the Portuguese version of brief COPE is a useful, time-efficient tool for both practitioners and researchers who need to assess coping strategies, a relevant construct in family context.

## 1. Introduction

Coping can be defined as the cognitive and behavioural efforts deployed to solve specific internal and/or external demands that are evaluated by the person as being excessive for their resources [1]. Studies on coping can be divided into those that define it as a stable trait and those that define it as a series of strategies which are implemented depending on the situation [2]. As for the latter, coping with stress should be studied independently of its results, since its effectiveness largely depends on the type of individual, threat, context and which results are required. Although most research has focused on the individual, the study of coping must incorporate dimensions related to the social context in which it is carried out, providing relevant information that helps to identify the causes of the emergence of strategies, their effectiveness, and their role in the consequences of stress [3]. According to Boss and colleagues [4], the individual’s environment, such as family circumstances, can promote the development of strategies considered adaptive to the environment, increasing their capacity to resist stress.

There are families in which there is a variable, but significant, degree of risk for the well-being of children, although the situation is not serious enough to require child out-of-home placement [5,6,7]. In this context, and from a situational perspective, one type of stress that stands out is the one associated with the exercise of parenting. Taking into consideration one of the instruments most frequently used in the international literature to assess this dimension, the Parenting Stress Index—Short Form [8], at-risk families’ scores generally fall between 80 to 100 points in the total parental stress scale [7,9,10,11], whereas families from community samples score around 70 points [9,12]. This indicates that parents living in contexts of psychosocial risk tend to frequently feel overwhelmed by the demands derived from their parenting role [10,11,13].

The study of coping strategies, therefore, entails the contextual and situational analysis of both the individual and the perceived demand, as well as the detailed description of the cognitions and coping behaviours that one tends to use. A large number of measurement scales have been created for this purpose. The vast majority of the extant instruments are related to the Ways of Coping Questionnaire by Lazarus and Folkman [14]. Different coping assessment tools have been developed, evaluating general (e.g., [15,16]) and specific stressful situations, such as work coping [17], chronic diseases [18], pain [19] or loss of a loved one [20].

Carver, Scheier and Weintraub [21] developed their Coping Orientation to Problems Experienced (COPE) questionnaire based on the transactional theory of stress [1] and the Model of emotional self-regulation [22]. Three types of coping were included (i.e., strategies focused on the problem, on emotion and avoidance), with 13 specific strategies. The different validation studies of this scale confirmed the suggested three-factor structure [23,24]. The main novelty of this instrument was that it could assess coping as a personal style or as a strategy related to a specific stressful situation.

Later, Carver developed a shorter version of the COPE (Brief COPE [25]), with a sample of 168 survivors of Hurricane Andrew. The author deleted two scales from the original instrument, added a new one (self-blame), and reduced each scale to two items, going from 60 items to 28 with a four-option Likert-type response format from “I do not do this at all” to “I usually do this a lot”. The analysis of the internal structure showed that this shortened version was similar to the full version, explaining 72.4% of the variance. The items were homogeneously distributed into 14 first-order subscales or strategies: active coping, planning, positive reframing, acceptance, humour, religion, using emotional support, using instrumental support, self-distraction, denial, venting, substance abuse, behavioural disengagement and self-blame. The religion, substance abuse, humour and venting scales, each constituted a unique factor.

Following a situational perspective, the Brief COPE has been administered to evaluate various stress strategies when facing different demands, as the study of stress management in parents of children with spinal cord injury [26], patients with head and neck cancer [27], patients with melanoma [28], survivors of different traumatic situations [29] or chronic patients with COVID-19 [30]. Moreover, the Brief COPE has been validated in different countries, such as France [31,32,33], Greece [34], USA [35], Spain [36] and Chile [37] obtaining, in general, satisfactory validity and internal consistency. 

In the Portuguese context, several validation studies have been carried out. Pais-Ribeiro and Rodrigues [38] developed the first Portuguese version with 364 university students. Four judges participated in the adaptation translation process and good internal consistency indices in the 14 scales were obtained. Maroco et al. [39] revised the version, in a sample of university students, with a different translation and back-translation process. A pre-test and post-test were carried out with the original and translated versions, where bilingual subjects participated. The validity of content and invariance between the Portuguese (448 students) and the Brazilian (1085 students) samples was satisfactory. However, a Brazilian-Portuguese adaptation of the instrument was recently done [40], in which consistency problems were identified in the subscales referring to first-order coping strategies.

Despite the Brief COPE potential usefulness, there are no validation studies with normative adult and at psychosocial risk Portuguese parents. The American Educational Research Association, the American Psychological Association, and the National Council on Measurement in Education [41] recommend collecting empiric evidence using assessment instruments with non-community populations. Hernández and colleagues [42] pointed out that the reliability and validity of standardized scales must be studied through repeated application of an instrument in diverse contexts and among different populations.

The aim of the present study was to examine the psychometric properties of the Brief COPE, extending its cross-cultural validity with a Portuguese sample of parents while attempting to address several of the other limitations of prior research. Specifically, this study attempts to validate its use among a community and at-risk sample of parents using factor analysis to examine the internal structure of the Brief COPE, testing for measurement invariance. We expected: (1) to confirm the 14-factor structure of the Brief COPE; (2) to show cross sample measurement invariance of the Brief COPE; (3) the Brief COPE to show good internal consistency measured by Cronbach’s alpha; and (4) to obtain significant relations with family adaptability, cohesion and parenting stress.

## 2. Materials and Methods

### 2.1. Participants

A community sample of 153 parents (M = 38.25 years; SD = 6.89 years; age range = 21–58 years) agreed to voluntarily participate in the study (78.3% women). They had a medium-high education level: 34.6% had completed secondary education and 30.7% had completed higher education studies. Their children had a mean age of 9.65 years old (SD = 4.99; Range: 1–18) and 52.3% were boys.

The at-risk sample included 116 parents of Child Welfare Services (CWS) referred children (M = 36.64 years; SD = 8.61 years; age range = 16–61 years). Most of them were women (80.2%) and had a low educational level: 43.1% had not completed compulsory education and 32.8% completed only primary education. Their children had a mean age of 10.22 years old (SD = 4.32; Range: 1–17) and 57.8% were boys.

No differences were found between the two samples regarding age (F = 2.09; *p* = 0.10) and gender distribution (*χ*^2^(1, *N* = 269) = 1.17, *p* = *n.s.*), but we found differences concerning educational level (*χ*^2^(3, *N* = 269) = 59.41, *p* = 0.000). Moreover, no differences were found between groups regarding child’ age (F = 0.99; *p* = *n.s.*) and gender (*χ*^2^(1, *N* = 269) = 0.80, *p* = *n.s.*).

### 2.2. Measures

#### 2.2.1. Brief Coping Orientation to Problems Experienced Inventory (Brief COPE)

As described previously, it is a self-response, multidimensional scale which can be used to evaluate both strategies and coping styles. To obtain a preliminary Portuguese version of the scale, a forward-backward translation strategy was adopted, with the collaboration of two translators with a background in psychology research. The cultural adaptation was particularly considered, taking into account clarity, common language use, and conceptual equivalence of the scale. Discrepancies were revised until no semantic differences were detected between the English version and the Portuguese version (i.e., the translated items had the same or very similar meanings to the original English items) [25].

#### 2.2.2. Parenting Stress Index—Short Form (PSI)

This is a self-report instrument with 36 items, answered using a 5-point range (1 = “Strongly Disagree” to 5 = “Strongly Agree”) and assesses three dimensions of stress associated with the parenting role: Parental Distress (PD), Parent-Child Dysfunctional Interaction (PCDI) and perception of the child as a Difficult Child (DC). We used the Portuguese version of Abidin and Santos [43]. Higher scores indicate greater distress associated with the exercise of parenting. The subscale Parental Distress quantifies the individual’s feelings of discomfort with the parenting role. The Parent-Child Dysfunctional Interaction subscale evaluates the extent to which the parent feels that the child meets the parent’s expectations and the way their interaction makes the parent feel. The Difficult Child subscale focuses on the child’s characteristics and behaviours that facilitate or restrain the parent. The minimum and maximum possible scores are 12-60 for each subscale and 36-180 for the PSI-SF total score. Internal consistency for the present study, estimated by Cronbach’s alpha, was α = 0.87 to PD, α = 0.83 to PCDI, α = 0.86 to DC, and α = 0.93 to PSI [8].

#### 2.2.3. Family Adaptability and Cohesion Scales (FACES III)

We used the Portuguese version of Curral et al. [44]. This 20 items scale assesses two dimensions of family functioning: cohesion (i.e., emotional ties between family members, measured by odd items, 10 items) and adaptability (i.e., the degree of flexibility that the family has to change rules and roles in order to respond to problems, measured by even items, 10 items). The items are answered on a scale of 5 points (1 = Never to 5 = Almost always), and higher scores correspond to higher levels of cohesion and adaptability. Internal consistency for the present study, estimated by Cronbach’s alpha, was α = 0.80 to cohesion and α = 0.62 to adaptability [45].

#### 2.2.4. Sociodemographic Questionnaire

A sociodemographic questionnaire was also applied to obtain data about participants’ age, gender, socioeconomic status, and years of schooling completed.

### 2.3. Procedures

Community sample: Using a snowball sampling technique, master students from the Psychology Department of the University of Algarve were contacted and asked to participate as intermediate by recruiting five parents each to answer the Brief COPE, FACES III and PSI.

At-risk sample: Mothers and fathers who fulfilled the selection criteria were asked to participate in the study by workers from Child Protection Services of Algarve (South of Portugal). Parent’s selection criteria were: (1) Being enrolled in CWS for family preservation reasons for at least three months and (2) Not facing a family crisis during recruiting and data collection. 

The research was approved by University of Algarve. Participants were informed about the aims of the research study, its non-compensatory nature, the anonymous and confidential nature of their responses as well as the possibility of withdrawing the study at any time without any negative consequences. No participants were excluded in either group.

The data were analysed using IBM SPSS 24.0 (IBM Corp., Chicago, IL, USA) and EQS 6.3 [46]. The factor structure of the Portuguese language version of the Brief COPE scale was assessed with Confirmatory Factor Analysis (CFA) performed with ML robust estimation methods [47]. Goodness of fit indices were calculated, including Satorra-Bentler chi-square/degrees of freedom, comparative fit index (CFI), incremental fit index (IFI), root mean square error of approximation (RMSEA) and Akaike Information Criterion (AIC). Regarding the incremental fit index, also known as Bollen’s IFI, values that exceed 0.90 were regarded as acceptable. In terms of the Akaike Information Criterion (AIC), which measures the expected discrepancy between the true model and the hypothesized model, the model with the smallest AIC should be selected [48]. The CFA was performed on the original scale items. No modification indexes were considered to improve the measurement model.

Measurement invariance was also evaluated. The *S-Bχ*^2^ difference test, the *Δ*CFI, and the *Δ*RMSEA were used to determine if the constraints significantly deteriorated the fit of the model [49]. ANOVAs were used to examine differences between the groups, including partial Eta squared (*η_p_^2^*) effect size [50]. Pearson correlations were used to analyse associations between scale variables.

## 3. Results

Our first step in examining the psychometric properties of the Brief COPE was to use CFA to replicate the different factor structures proposed for this instrument. We found strong support for the 14-factor model based on appropriate goodness-of-fit indices [48] (see Table 1).

Table 2 displays the standardized item loadings for the 14-factor model structure estimate with the ML Robust method. In the community sample, most items had loadings well above 0.40, except factors 5, 12 e 13. In the at-risk sample, most items loaded above 0.30 except factors 5, 10 and 11. None of the items were removed from the model.

The next step was to test for measurement invariance across samples (community versus at-risk) using the 14-factor model. The configural model (i.e., no constrains included) served as the baseline against which to compare stricter models. That is, the fit indices of the baseline model were compared with a model where factor loadings are equally constrained across groups (i.e., weak or metric invariance) and with a model where factor loadings and covariances are equally constrained across groups (i.e., strong or scalar invariance), which are shown in Table 3.

Regarding cross samples invariance, results showed that the Δ*S-Bχ2*(*df*) values were significant in terms of week and strong invariance, but if we consider Cheung and Rensvold´s [49] criteria (i.e., ΔCFI not exceeding 0.01, *Δ*RMSEA less than 0.015) and standard criteria regarding CFI and RMSEA (i.e., CFI above 0.90, RMSEA below 0.08) there is support for measurement invariance across samples.

Table 4 presents Pearson correlations between Brief COPE dimensions among at-risk and community sample.

Table 5 displays the alphas, the mean inter-item correlations, and the corrected item-total correlation range for the Brief COPE and its dimensions among the two samples. The results were mostly good or acceptable, although many of the mean inter-item correlation were above the recommended value of 0.50 [51]. The community sample shows higher scores in most subscales, except in using emotional support and behavioural disengagement.

Table 6 displays the correlations between the Brief COPE factors, FACES and PSI dimensions. As expected, most adaptive coping strategies show positive associations with family adaptability and cohesion, and negative associations with parenting stress. Maladaptive coping strategies show a positive and significative relation with parenting stress. Planning, Religion and Substance Use did not show significative relations with any dimension of family functioning or parenting stress.

Regarding group validity, Table 7 displays comparisons between community and at-risk participants.

Although significant differences were observed between both groups in almost all dimensions, they had a small effect size, except for the differences in Active coping, Denial and Self-blame which a moderate effect size. In these dimensions, at-risk sample presented higher values. Regarding concurrent validity, we observed a negative and significative association between parents’ age and Using Instrumental Support (*r* = 0.16, *p* = 0.01), Venting (*r* = 0.13, *p* = 0.04) and Behavioural Disengagement (*r* = −0.15, *p* = 0.01).

## 4. Discussion

The Brief COPE (28 items [25]) was developed to deal with some constrains (e.g., impatience, difficulty in completing) felt by participants with the full COPE, but maintained its original 14-scales, using the items with higher clarity and greater factor loadings. This COPE short version has been applied in different contexts (e.g., Spain [36]; Chile [37]; New Zealand [52]) and with diverse samples (e.g., adults, caregivers for a family member living with adults with HIV/AIDS, adults with mild traumatic injury) [40], but as we mentioned, psychometric studies purposively involving parents have not been conducted so far, to the best of our knowledge.

Although the extant research has shown satisfactory evidence of validity and internal consistency, some authors had considered COPE as unstable and with results somewhat dependent on the analysis method used [40]. Being consistent with another Portuguese validation study [39], we used CFA analysis to replicate the different factor structures proposed for this instrument—an unifactorial and a 14-factor model in both samples (i.e., at-risk and community parents) was tested. Our results presented appropriate goodness-of-fit indices for the 14-factor solution and, as most of the items had loadings above 0.40 (mainly in the community sample) we decided to maintain all the items in the model. Other studies have found support for this structure [33]. The findings of the configural model in both samples (i.e., at risk and community) reveal invariance across samples, which suggests that the instrument is suitable to evaluate parental coping both in community and in at-risk populations.

The inter-dimensions correlations were higher in the community sample than in the at-risk sample, and we found significant negative correlations between behavioural disengagement and active coping and positive correlations between behavioural disengagement and self-distraction in the at-risk sample. This shows that vulnerable parents tend to use fewer positive strategies, encompassing initiative to solve the stressful situation, and more avoidance and escapist strategies, which can be somewhat analysed in light of approach-avoidance theories [53]. Not only do parents from at-risk contexts feel overwhelmed by parenting role demands [10,13], but they also reveal adopting fewer and not as effective coping strategies. As Liga and colleagues [54] pointed out, when some people with vulnerability features experience strong pressure, they can perceive their skills for dealing with the situation as limited and end up choosing avoidant strategies [55,56].

Regarding internal consistency, the results were mainly good or acceptable, mainly in the community sample. However, there are some limitations, in general in the at-risk sample, which can be linked to the sample size. The Brief COPE has 14-dimensions and 28-items, leaving two-items per dimension and also a participant-per-item ratio which is inferior to the recommended number. However, our results are consistent with other 14-factors studies [25,32]. This instrument, according the original author [25], is not meant to provide an overall score, but instead 14-dimensions independent scores, which is deemed to be more useful for research and intervention purposes.

As expected, the more adaptive factors of Brief COPE were positively correlated with family adaptability and cohesion, and maladaptive factors were positively correlated with parenting stress. These results confirm the convergent and divergent validity of this instrument, showing that positive (e.g., cohesion and adaptability) and negative aspects (e.g., stress) of the familial and extra-familial social relationships are associated to the individual’s coping strategies [4].

The differences in coping strategies that we observed between the two samples (i.e., at-risk and community), although significative, had a small effect size, so we can assume that this research validates the Brief COPE as an important tool for assessing parents’ coping strategies in the Portuguese context.

The at-risk group obtained higher scores in active coping, denial and self-blame, and the youngest parents were the ones who reported using more instrumental support, venting and behavioural disengagement. These findings may indicate that parents’ vulnerabilities (e.g., lower income, basic educational levels, parenting inexperience) can cause negative emotions and behaviours [57], especially when there are fewer environmental resources (e.g., social support, education, positive reframing). The at-risk group reported a lower educational level compared to the community sample. This result is similar to other studies that showed that at-risk families live in precarious economic, employment and educational conditions and therefore constitute a vulnerable group [6,7,11].

Vulnerable parents have also been reported to show greater difficulties in managing stress situations, which may compromise the development of other family members [58,59], their social functioning and their quality of life [57,60]. These assumptions can be used by professionals, who play an important role in promoting adaptative coping strategies in positive parenting programs [61].

Moreover, a note of caution is needed due of the items’ loadings in some of the 14-factors (mainly in the at-risk sample), which should be further analysed in future studies. Concerning the psychometric procedures, a larger sample is also necessary, in which other assumptions for a confirm validation assessment must be provided.

Our research addressed the evaluation of the coping style and strategies of a specific population and assessed the Brief COPE [25], finding appropriate psychometric properties.

## 5. Conclusions

Our main results support the notion that Brief COPE may be a useful tool for assessing psychosocial needs and problems of parents. The Brief COPE has shown satisfactory validity and reliability properties to be used in the assessment of Portuguese parents’ coping strategies from both community and at-risk populations. These findings reinforce the usefulness of this measure in pre- and post-test for evidence-based parenting interventions, as well as for in-service training and social work education.

## Figures and Tables

**Table 1:** Goodness of Fit Indices for ML Models.

Brief COPE	S-B*χ*^2^/*df*	IFI	NNFI	CFI	RMSEA	Confidence Interval (90%)	AIC
**At-risk sample**							
Unifactorial Model	3.22	0.43	0.38	0.42	0.14	0.13–0.15	426.74
14-Factor Model	1.06	0.99	0.98	0.99	0.02	0.00–0.04	−244.01
**Community sample**							
Unifactorial Model	3.36	0.82	0.81	0.82	0.12	0.12–0.13	476.51
14-Factor Model	1.37	0.98	0.97	0.98	0.05	0.04–0.06	−162.79

Note. ML = Maximum likelihood; S-B*χ*^2^ = Satorra-Bentler Chi-Square; *df* = Degrees of Freedom; IFI = Incremental Fit Index; CFI = Comparative Fit Index; NNFI = Non-Normed Fit Index; RMSEA = Root Mean Square Error of Approximation; AIC = Akaike Information Criterion.

**Table 2:** Standardized factor loadings of the 14-factor model of Brief COPE (at-risk sample/community sample).

Brief COPE	F1	F2	F3	F4	F5	F6	F7	F8	F9	F10	F11	F12	F13	F14
Item 2	0.34/0.56													
Item 7	0.41/0.60													
Item 14		0.52/0.74												
Item 25		0.33/0.76												
Item 12			0.25/0.63											
Item 17			0.49/0.49											
Item 20				0.51/0.61										
Item 24				0.43/0.74										
Item 18					0.22/0.33									
Item 28					0.10/0.20									
Item 22						0.51/0.44								
Item 27						0.21/0.40								
Item 5							0.48/0.55							
Item 15							0.52/0.67							
Item 23								0.51/0.64						
Item 10								0.36/0.75						
Item 1									0.34/0.40					
Item 19									0.15/0.47					
Item 3										0.21/0.44				
Item 8										0.26/0.42				
Item 9											0.29/0.50			
Item 21											0.22/0.57			
Item 4												0.30/0.20		
Item 11												0.41/0.25		
Item 6													0.23/0.16	
Item 16													0.29/0.16	
Item 13														0.19/0.49
Item 26														0.30/0.41

Note. F = Factor.

**Table 3:** Tests for invariance of Brief COPE goodness of fit statistics.

Model	S-B*χ*^2^ (*df*)	∆S-B*χ*^2^ (*df*)	* CFI (ΔCFI)	* RMSEA (ΔRMSEA)
Cross sample (at-risk /Community)				
1. Configural model (no constrains)	629.70 (518)	-	0.98 (-)	0.04 (-)
2. Weak (metric) invariance	665.96 (532)	33.63 (14) **	0.98 (0.00)	0.04 (0.00)
3. Strong (scalar) invariance	808.75 (637)	172.44 (119) ***	0.97 (0.01)	0.05 (0.01)

Note. S-Bχ^2^(*df*) = Satorra-Bentler chi-square (degrees of freedom); * CFI = robust Comparative Fit Index; * RMSEA = robust Root Mean Square Error of Approximation; ** p < 0.01; *** p < 0.001.

**Table 4:** Brief COPE correlation matrixes between its dimensions among community (*n* = 136) and at-risk sample (*n* = 116).

Dimensions	1	2	3	4	5	6	7	8	9	10	11	12	13	14
1. Active Coping	-	0.67 ***	0.52 ***	0.51 ***	0.12	0.30 ***	0.47 ***	0.44 ***	0.21 **	0.25 **	0.40 ***	−0.03	−0.00	0.20 *
2. Planning	0.55 ***	-	0.59 ***	0.69 ***	0.17 *	0.32 ***	0.57 ***	0.54 ***	0.36 ***	0.30 ***	0.50 ***	0.15	0.06	0.38 ***
3. Positive Reframing	0.46 ***	0.35 ***	-	0.63 ***	0.29 ***	0.29 ***	0.43 ***	0.37 ***	0.35 ***	0.22 **	0.30 ***	0.04	−0.10	0.07
4. Acceptance	0.35 ***	0.34 ***	0.53 ***	-	0.35 ***	0.34 ***	0.49 ***	0.45 ***	0.38 ***	0.27 **	0.42 ***	0.16	0.12	0.27 **
5. Humour	0.12	0.01	0.13	0.19 *	-	0.10	0.20 *	0.18 *	0.10	0.17 *	0.14	0.22 **	0.03	0.01
6. Religion	0.08	0.15	0.17	0.23 *	−0.02	-	0.32 ***	0.33 ***	0.24 **	0.32 ***	0.26 **	0.04	0.17 *	0.19 *
7. Using Emotional Support	0.23 *	0.26 **	0.30 **	0.33 ***	0.20 *	0.10	-	0.75 ***	0.33 ***	0.25 **	0.40 ***	0.16	0.08	0.33 ***
8. Using Instrumental Support	0.23 *	0.30 **	0.18	0.29 **	0.05	0.10	0.71 ***	-	0.40 ***	0.41 ***	0.56 ***	0.19 *	0.14	0.38 ***
9. Self-Distraction	−0.03	0.08	0.16	0.23 *	−0.07	−0.07	0.07	0.13	-	0.51 ***	0.47 ***	0.26 **	0.26 **	0.42 ***
10. Denial	−0.03	0.08	−0.06	−0.02	−0.15	−0.03	−0.03	0.06	0.30 **	-	0.46 ***	0.27 **	0.42 ***	0.49 ***
11. Venting	−0.03	0.09	−0.06	−0.12	−0.04	0.07	0.22 *	0.12	0.22 *	0.24 **	-	0.22 **	0.26**	0.60 ***
12. Substance Use	−0.04	0.07	0.07	0.03	−0.14	0.30 **	−0.02	0.11	0.01	0.02	0.22 *	-	0.18 *	0.28 ***
13. Behavioural Disengagement	−0.20 *	−0.07	−0.13	−0.01	−0.14	0.09	−0.12	−0.11	0.34 ***	0.34 ***	0.24 **	0.12	-	0.30 ***
14. Self-Blame	0.04	0.10	0.03	0.16	−0.04	0.13	−0.09	−0.02	0.28 **	0.31 **	0.14	0.13	0.24 **	-

Note. * *p* < 0.05, ** *p* < 0.01, *** *p* < 0.001.

**Table 5:** Internal consistency of Brief COPE.

At-Risk Sample/Non-Risk Sample	Alpha	MIIC	CITCR
Active Coping	0.67/0.69	0.52/0.53	0.52/0.53
Planning	0.57/0.80	0.40/0.66	0.40/0.66
Positive Reframing	0.44/0.70	0.28/0.53	0.28/0.53
Acceptance	0.63/0.74	0.46/0.59	0.46/0.59
Humour	0.80/0.81	0.67/0.69	0.67/0.69
Religion	0.72/0.82	0.56/0.70	0.56/0.70
Using Emotional Support	0.81/0.61	0.68/0.44	0.68/0.44
Using Instrumental Support	0.72/0.83	0.56/0.71	0.56/0.71
Self-Distraction	0.37/0.43	0.23/0.28	0.23/0.28
Denial	0.59/0.62	0.42/0.45	0.42/0.45
Venting	0.46/0.46	0.30/0.30	0.30/0.30
Substance Use	0.88/0.88	0.79/0.79	0.79/0.79
Behavioural Disengagement	0.80/0.57	0.67/0.40	0.67/0.40
Self-Blame	0.68/0.77	0.52/0.62	0.52/0.62

Note. Alpha = Cronbach’s alpha, MIIC = mean inter-item correlation, CITCR = corrected item-total correlation range.

**Table 6:** Brief COPE correlation matrixes between its dimensions and family cohesion, adaptability and parenting stress (community/at-risk sample).

Brief COPE Factors	FACES Cohesion	FACES Adaptability	PSI Total
Active Coping	0.15/0.35 ***	−0.04/0.25 **	0.06/−0.26 **
Planning	0.12/0.14	0.07/0.18	0.07/−0.14
Positive Reframing	0.13/0.33 ***	0.11/0.29 **	0.07/−0.20 *
Acceptance	0.10/0.25 **	0.07/0.20 **	0.17 */−0.10
Humour	0.31 ***/0.17	0.24 **/0.20 **	0.26 **/−0.05
Religion	0.11/0.01	−0.01/−0.02	0.07/0.07
Using Emotional Support	0.26 **/0.31 **	0.18 */0.16	0.04/−0.05
Using Instrumental Support	0.19 */0.15	0.08/0.08	0.10/0.01
Self-Distraction	0.06/−0.07	0.14/0.12	0.24 **/0.25 **
Denial	0.03/−0.20 *	−0.06/−0.06	0.14/0.28 **
Venting	0.10/−0.06	0.02/0.04	0.25 **/0.30 **
Substance Use	0.09/−0.05	0.18 */−0.08	−0.10/0.02
Behavioural Disengagement	−0.19 */−0.19 *	−0.11/0.07	0.18 */0.28 **
Self-Blame	0.05/−0.14	0.09/0.05	0.32 ***/0.29 **

Note. * *p* < 0.05, ** *p* < 0.01, *** *p* < 0.001.

**Table 7:** Brief COPE comparison among community and risk sample.

Dimensions	At-Risk Sample	Community Sample	F	*η* ^2^
	M (SD)	M (SD)		
Active Coping	7.11 (1.09)	6.39 (1.71)	15.69 ***	0.06
Planning	6.76 (1.18)	6.14 (1.74)	10.71 **	0.04
Positive Reframing	5.71 (1.58)	5.44 (1.75)	1.61 *^ns^*	0.01
Acceptance	6.07 (1.51)	5.51 (1.79)	7.35 **	0.03
Humour	3.34 (1.71)	4.03 (1.93)	9.49 **	0.04
Religion	4.02 (2.05)	3.96 (1.99)	0.05 *^ns^*	0.00
Using Emotional Support	5.65 (1.90)	5.05 (1.75)	7.07 **	0.03
Using Instrumental Support	5.75 (1.74)	5.22 (1.94)	5.32 *	0.02
Self-Distraction	4.76 (1.86)	3.99 (1.65)	12.87 ***	0.05
Denial	4.91 (1.93)	3.82 (1.69)	23.90 ***	0.09
Venting	4.65 (1.70)	4.28 (1.66)	3.14 *^ns^*	0.01
Substance Use	2.60 (1.33)	2.14 (0.80)	12.38 **	0.05
Behavioural Disengagement	2.79 (1.15)	2.62 (1.15)	1.18 *^ns^*	0.00
Self-Blame	5.43 (1.84)	4.20 (1.84)	28.25 ***	0.11

*Note*. *ns*- non-significant, * *p* < 0.05, ** *p* < 0.01, *** *p* < 0.001.

## Data Availability

Data can be available to consultation when request to the corresponding author and with per-mission of the participants of the study.
